# Workplace Harassment and All-Cause Mortality in a Longitudinal Cohort over a 24-Year Period

**DOI:** 10.3390/occuphealth1020021

**Published:** 2026-06-01

**Authors:** Kathleen M. Rospenda, Sally Freels, Timothy P. Johnson, Judith A. Richman

**Affiliations:** 1 Department of Psychiatry, University of Illinois Chicago, 1601 W. Taylor St., Chicago, IL 60612, USA; 2 Division of Epidemiology and Biostatistics, School of Public Health, University of Illinois Chicago, 1603 W. Taylor St., Chicago, IL 60612, USA; 3 NORC at the University of Chicago, 55 East Monroe St., 30th Floor, Chicago, IL 60603, USA

**Keywords:** workplace harassment, sexual harassment, psychosocial stressors, worker health

## Abstract

The objective of this research was to examine the effects of sexual and generalized harassment in the workplace on risk for all-cause mortality in a sample (*n* = 1745) originally drawn from a university workplace and followed over a 24-year period after baseline. Eleven timepoints of data on self-reported workplace harassment were collected between October 1996 and February 2021, at time intervals ranging from one year to 13 years, and linked to mortality data (*n* = 249 deaths) from the National Death Index through December 2021. We used proportional hazards modeling to examine the risk for all-cause mortality associated with workplace harassment (as measured by a modified version of the Sexual Experiences Questionnaire and the Generalized Workplace Harassment Questionnaire) occurring in the previous time period. We also examined differential risk by gender for White and Black study participants. In fully adjusted models, experiencing generalized harassment (GH) was associated with significantly increased hazard of mortality at the next time point for White women (HR = 1.03, *p* < 0.01). Experiencing sexual harassment (SH) was associated with a trend-level increase in the hazard of next-time-point mortality for Black women (HR = 1.05, *p* = 0.09). Neither SH nor GH was associated with increased hazard of mortality for men. Workplace interventions to address harassment, stronger enforcement of sexual harassment policy and law, and enactment of policy and law to prevent generalized harassment and bullying may contribute to the reduction of all-cause mortality among working women.

## 1. Introduction

Over the past decade, there has been increasing attention to the relevance of psychosocial workplace stressors for worker health. Psychosocial work stressors or hazards are factors related to the design, management, or social context of work that can negatively impact the health of workers and, in fact, as researchers have recently argued, may soon surpass other types of workplace hazards as a risk factor for ill health [1]. Research has also found that the impact of psychosocial hazards on health can extend to mortality; however, such research has generally been limited to testing components of the Job Strain Model which posits that the combination of high job demands (e.g., having too much work, or not enough time to complete work) and low job control leads to job strain [2]. A systematic review and meta-analysis of research on the link between psychosocial hazard exposure and all-cause mortality concluded that low job control (i.e., lack of control over workplace decisions), but not high job demands, job strain (a combination of high demands and low control), shiftwork, or job insecurity, was associated with increased hazard of all-cause and coronary heart disease mortality [3].

Workplace harassment (WH) is a psychosocial hazard that is associated with symptoms of poor health and problematic health-related behaviors (e.g., alcohol misuse) but has not been included in prospective studies of mortality. While workplace harassment might conceptually be considered to be a psychological job demand, it is distinct from the actual job demand construct as measured in the Job Strain Model, which is limited to demands involving the quantity and pace of work (i.e., having to work hard or fast, not having time to get the job done, being asked to do an excessive amount of work, having conflicting demands, having long periods of concentration, waiting for work on other people, having a hectic job) and low control over how work is done [2]. By contrast, workplace harassment conceptually involves various ways that one can be mistreated on the job, which is a qualitatively different experience from having demands on the quantity and/or pace of work.

Two commonly studied forms of workplace harassment are sexual harassment (SH)—unwelcome sexual conduct that affects an individual’s job status or creates a hostile/offensive work environment [4]—and generalized harassment (GH)—interpersonal mistreatment not based on gender or other legally protected characteristics (e.g., race/ethnicity, age). Both of these forms encompass experiences such as verbal aggression, disrespectful or exclusionary behavior, and threats/bribes. GH is a construct which is similar to workplace bullying, without the requirement for repeated harassment over a specific timeframe [5]. In the only national study of the prevalence of SH and GH in the United States, researchers found that approximately 50% of women and up to 40% of men reported having ever experienced at least one sexually harassing behavior, and about 60% of both women and men reported at least one behavior aligning with GH in the past 12 months [6]. These estimates are consistent with a recent meta-analysis of WH prevalence [7]. Applying these estimates to the current U.S. civilian workforce [8] suggests that more than 102 million workers may have experienced some form of workplace harassment in the past year.

Empirical research clearly demonstrates the public health relevance of exposure to workplace harassment (WH). Both SH and GH are associated with a variety of negative mental and behavioral health outcomes, including psychological distress and alcohol use [6,9], as well as symptoms of poor physical health [10,11]. Sexual harassment in particular has been linked to increased risk for hypertension and cardiovascular disease among women [12–14], with some evidence that this association may differ by race, with White women particularly at risk of increased blood pressure associated with SH [13]. GH is associated with increased odds of cardiovascular disease for both men and women [15]. Given that cardiovascular disease is the leading cause of death in the United States [16], it follows that SH and GH may increase the risk for mortality.

One question is whether workplace harassment poses greater health and mortality risks for women than for men. Research on other job stressors supports possible gender differences in mortality risk associated with job stressor exposure. Indeed, available research suggests that some aspects of the workplace environment may more severely impact the health of male employees, possibly by directly threatening male gender roles. In an Australian retrospective cohort study, low job control was associated with a staggering 81% increase in the risk of mortality over a 15-year period among male participants, but there was no comparable increase in risk for female participants [17]. Similarly, a separate meta-analysis of the association between job strain and mortality found increased risk only for men [18]. In contrast, it is possible that sexual harassment may be more strongly associated with increased risk for mortality among women, as it is a stressor both experienced more frequently by and associated with increased health risks for women. However, research has not yet investigated this possibility. Moreover, the “double jeopardy” hypothesis [19] suggests that, as members of two disadvantaged social groups defined by gender and race/ethnicity, minority women may be at particularly increased risk of both exposure to and negative effects associated with stressors such as workplace harassment, possibly including increased risk for mortality, although this too has yet to be tested.

This study aims to fill a gap in the research on workplace psychosocial hazards and mortality by examining the association between exposure to GH and SH and all-cause mortality over a 24-year period. We hypothesize (1) that exposure to SH and/or GH will be associated with increased risk for mortality and (2) that the association between SH and mortality will be stronger for women than for men, based on existing research [12–14] demonstrating increased risk for cardiovascular disorder associated with SH among women. The existing literature does not suggest a clear hypothesis regarding possible differences in the risk of mortality associated with GH for men versus women, so we explore this as a research question. We also explore (1) whether the risk for mortality associated with SH and GH may differ by race as well as gender, hypothesizing that Black women, given their doubly disadvantaged social status, will experience the highest risk for WH-related mortality, and (2) whether the inclusion of concurrent symptoms of depression and alcohol use/misuse alters any observed effects of WH on mortality, given prior research demonstrating associations between exposure to WH and depressive symptoms [20,21] and alcohol use/misuse [6,22], and associations of both depression [23,24] and alcohol misuse [25–27] with increased risk for morbidity and mortality.

## 2. Materials and Methods

**Figure 1.**
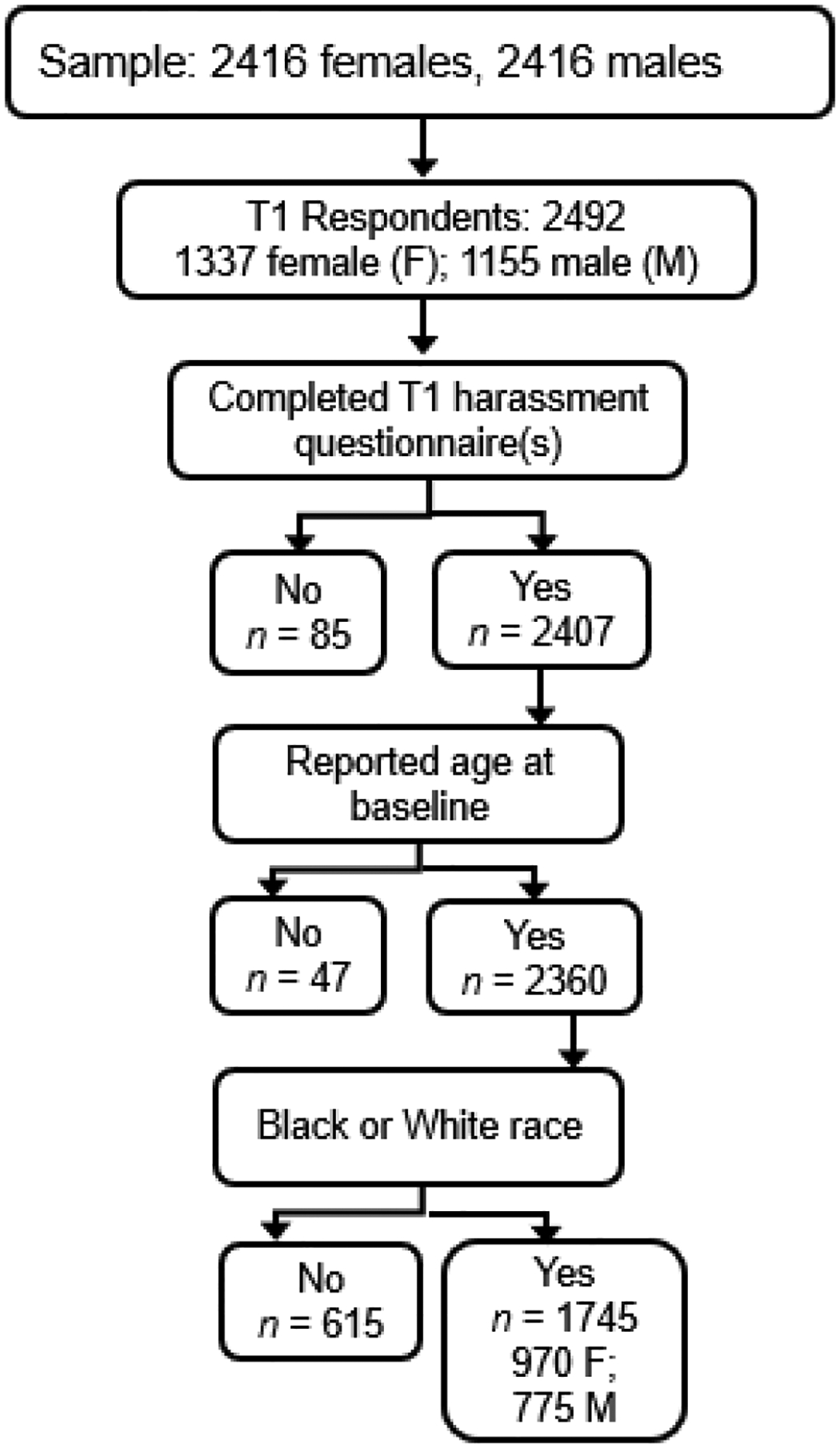
Flowchart for study sample. Excludes *n* = 179 who only responded at T1 and did not die during follow-up, and *n* = 7 with problematic survey data.

The data derive from a longitudinal cohort study of individuals originally sampled and recruited in 1996 from an urban, Midwestern university workplace and surveyed on their experiences of workplace harassment for eleven time points over nine data collection waves through to February 2021 (T1–T9). Participants were sampled from four occupational groups—clerical/administrative, faculty, graduate student workers (graduate research assistants, medical residents, postdocs), and service/maintenance workers—stratified by male/female gender (2416 males, 2416 females), and were sent mail surveys with postage-paid return envelopes. A total of 2492 individuals responded at T1, between October 1996 and February 1997 (American Association for Public Opinion Research response rate formula RR2 = 52% [28]). Participants were resurveyed at approximately 1-year intervals between T1 and T7, with the exception of a three-year gap between T2 and T3, and a 2-year gap between T5 and T6. Participants were asked to retrospectively report on their harassment experiences during those gaps. The T8 questionnaire in 2008 did not inquire about workplace harassment experiences. Prior survey participants were resurveyed about their workplace harassment experiences at T9 (June 2020–February 2021). Thus, the survey period covered approximately 24 years, with eleven years of exposure data for workplace harassment. The incentives for survey participation were $20 at T1 and T2, $30 at T3 through T7, and $50 at T9. National Death Index records were searched for death dates and causes for the years 1996 through 2021 for study participants. We excluded from consideration those who only responded at T1 and did not die during follow-up (*n* = 179) or had problematic data (survey responses received after death date) (*n* = 7). Analyses for this paper further excluded individuals (1) if data were missing on age at baseline (T1), (2) if data regarding T1 SH or T1 GH were missing, or (3) if the respondent race/ethnic group was other than White or Black, as these groups individually had insufficient size for meaningful analysis. The flowchart for the study sample is shown in Figure 1. Further details about the study design and measures are reported in prior publications [29,30].

Workplace sexual harassment experiences in the past 12 months were measured at T1–T7 and T9 using a modified version of the 19-item Sexual Experiences Questionnaire [31], with wording modified to apply to either women or men. Respondents indicated the frequency of experience of gender harassment (6 items; e.g., told suggestive stories or offensive jokes), unwanted attention (6 items; e.g., unwanted attempts to stroke or fondle you), sexual coercion (6 items; e.g., implied faster promotions or better treatment if you were sexually cooperative), and sexual assault (1 item). Responses were made using a 3-point scale (0 = never, 1 = once, or 2 = more than once). Items were summed to create a composite scale score. The coefficient alpha reliability of this measure was 0.80 or higher at each data collection point.

Generalized workplace harassment experiences in the past 12 months were measured T1–T7 and T9 with the Generalized Workplace Harassment Questionnaire, a 29-item measure that assesses experiences of verbal aggression (9 items; e.g., yelled or screamed at you), disrespect (9 items; e.g., humiliated or belittled you in front of others), isolation/exclusion (5 items; e.g., ignored you or your work contributions), threats/bribes (3 items; e.g., offered you a subtle or obvious bribe to do something that you did not agree with), and physical aggression (3 items; e.g., threw something at you) [5]. Responses were made using a 3-point scale (0 = never, 1 = once, or 2 = more than once). Items were summed to create a composite scale score. The coefficient alpha reliability of this measure was 0.90 or higher at each data collection point.

### 2.1. Mortality

National Death Index records were searched through to 31 December 2021 for date and cause of death. The 2021 Death Index was matched against our study sample to identify all deaths during the study period and corresponding death dates. Anyone who did not appear in the 2021 Death Index was assumed to still be alive in 2021.

### 2.2. Covariates

Occupational group at baseline was part of the design of the study and was included as a covariate. The four groups were faculty, clerical/administrative, student workers (e.g., GRAs, TAs, medical residents), and service/maintenance. Current age was included as a time-dependent covariate. Education level and household income (income was collected only at T9) overlapped substantially and significantly with occupational group and, as such, were not included as covariates to avoid problems with collinearity. Depressive symptoms in the past 7 days, as measured by 7 items from the Center for Epidemiologic Studies Depression scale (CESD; coefficient alpha reliability was ≥0.82 at T1–T9) [32]; number of alcoholic drinks consumed in a typical day when drinking [33]; and alcohol misuse as measured by the Brief Michigan Alcoholism Screening Test (BMAST) [34] were also included as covariates in supplementary analyses.

### 2.3. Statistical Analysis

For survival analysis, complete observations are years from study baseline until death for those who died, and right-censored observations are years from study baseline until 2021 for those who did not die. Those missing T1 SH or GH data were excluded from analyses because survival analysis requires either complete observations of years from study baseline until death (for those who died) or years from study baseline until 2021 (for those who did not die; i.e., right-censored observations). Because WH, especially SH, is known to differ by gender and race/ethnicity [6], we examined the association of WH with mortality by race and sex (male/female; data on sex were collected at T1, and we did not assess gender identity). Initial analyses indicated significant effects of sex and race and significant interactions between effects of harassment and both sex and race. Small numbers of deaths for many of the racial categories precluded meaningful analysis, so we chose to analyze only Black and White subjects, allowing for examination of unique effects in the four categories crossing sex and race.

Proportional hazards multiple regression models were used to model the hazard of death at 1 through 23 years after baseline. Occupational group at baseline was included as a fixed covariate. Current age was included as a time-dependent covariate, calculated from the reported age at baseline and years between the baseline and follow-up waves. Time-dependent covariates reflecting repeated measurements during the previous year included workplace sexual harassment (SH) and generalized workplace harassment (GH). Across repeated surveys, if a time-dependent covariate was not reported, the last previous reported value was used (“last value carried forward”).

We also tested models incorporating concurrent depression and alcohol use as covariates to examine whether and how inclusion of these variables impacted any observed associations between WH and mortality.

## 3. Results

Table 1 is a summary of death rates and age and occupation distributions at study baseline for Black females, White females, Black males, and White males.

Table 2 shows the results of proportional hazards multiple regression models predicting the hazard of death as a function of prior-wave workplace harassment in two separate models: one for sexual (SH) and one for generalized (GH) harassment. Each model was adjusted for common effects of current age and occupation group at baseline and allowed for unique estimates of harassment effects for each of the four subgroups. Note that the sample sizes in Table 2 are smaller than those in Table 1 due to subjects missing SH or GH measures at baseline. The results suggest a significant association between GH and the hazard of death for White females (HR = 1.033, *p* = 0.0027) and a trend-level association between SH and the hazard of death for Black females (HR = 1.048, *p* = 0.0889).

In models adjusted for current symptoms of depression and alcohol use, including these covariates did not significantly alter the coefficients for WH (see Tables A1–A3); for simplicity, they are excluded from the final models reported in Table 2.

## 4. Discussion

To our knowledge, this is the first study to examine the associations between two forms of workplace harassment and all-cause mortality in adults. While the magnitudes of the associations were relatively weak, our findings are the first to indicate significantly elevated risk for mortality among White women experiencing generalized harassment. While Black women experienced elevated risk of all-cause mortality associated with sexual harassment, these findings were reduced to trend-level significance in models across race and gender and adjusting for covariates. Experiences of WH were not associated with all-cause mortality for men. These findings are consistent with prior research that has found SH to be associated with cardiovascular disease (CVD) among women at midlife [12] and extends these findings to demonstrate a trend-level association between SH and all-cause mortality for Black women specifically. This provides some tentative evidence for the “double jeopardy” hypothesis proposing that the association between workplace harassment and health may be more substantial for Black versus White women. While the findings for generalized harassment did not support the double jeopardy hypothesis, the effect size of the association was similar for Black and White women, suggesting a relative lack of power in the Black female group due to its lower sample size. It is possible that the double jeopardy hypothesis was not supported for GH for Black women because Black women may be more accustomed to being treated in generally disrespectful ways (i.e., GH) and more likely to take it for granted. By contrast, SH may be viewed as more culturally offensive and thus may continue to be associated with deleterious health outcomes. Qualitative research in this area would be helpful to better understand how Black women view and cope with experiences of GH versus SH. The associations between exposure to different forms of harassment and health outcomes for different demographic groups are also an important avenue of future study, given the possible contribution of workplace psychosocial stressors to health disparities. Unfortunately, one limitation of our study was that our sample was not large enough to examine the associations between WH and specific causes for mortality. Further long-term, longitudinal research with larger, more representative samples is needed to test the generalizability of our findings, and to examine whether CVD is the primary underlying factor associated with mortality among women exposed to WH.

It is possible that depression or substance misuse associated with WH may subsequently increase the risk for mortality either directly or indirectly by contributing to chronic health conditions. Although formal tests of mediation were not possible within the analytic framework of this study, we tested models that included current depressive symptoms, alcohol use, and alcohol misuse (see Appendix A Tables A1–A3). While these variables, particularly depressive symptoms, exhibited significant associations with mortality, neither the magnitude nor the significance of associations between WH and mortality was meaningfully affected by including these factors in our models.

Further research is needed to clarify pathways through which occupational stressors such as harassment are associated with increased risk for mortality. In their review of biological mechanisms underlying the link between exposure to stressors and disease, Bagby and colleagues cite evidence for several other potential mechanisms, namely changes in organ structure and function, and disruption of the hypothalamic pituitary axis (HPA), immunologic, metabolic, and/or neurologic systems. These pathways culminate in three direct mediators of the stressor exposure–disease relationship—inflammation, oxidative stress, and insulin resistance—which contribute over time to the development of chronic disease [35], ultimately increasing the risk for mortality. Future research combining assessments of workplace harassment with biological indicators such as cortisol and markers of inflammation over time could help clarify which biological pathways are central to the association between WH and mortality.

Additionally, it is possible that exposure to workplace harassment may lead to the proliferation of other stressors that may negatively impact health, such as job loss and subsequent financial difficulties. Indeed, research found that prior exposure to chronic generalized workplace harassment was associated with greater exposure to current life stressors, including lower income, over 20 years after baseline, and that current stressors mediated associations between prior harassment exposure and current mental health symptoms and alcohol misuse [29]. Future longitudinal research comprehensively measuring life and job stressors such as workplace harassment could help clarify the causal pathway of stressor proliferation, leading to a more complex understanding of the way in which different patterns of stressor exposure over one’s lifetime contribute to health and health disparities.

Another limitation of this study was the longer gaps in measurement between T1 and T8 that may have led to inaccurate recall of harassment experiences. Similarly, the large gap in measurement between T8 and T9 likely negatively impacted study retention, with participants increasingly likely to be lost to follow-up. Future research would benefit from employing more consistent and frequent measurement points. This would enable better assessment of the specific timing of exposure to workplace harassment and its potential relation to any subsequent changes in health-related behaviors and/or biomarkers and how these changes contribute to disparities in health and mortality over time.

## 5. Conclusions

Our findings build on prior research on occupational risk factors for mortality by demonstrating associations between SH and GH and all-cause mortality. Although the findings are relatively weak, their significance lies in the fact that workplace harassment is both a prevalent and preventable occupational risk factor. These results support the importance of establishing strong and enforceable workplace policies, as well as enforcing legislation that prohibits workplace sexual harassment, to better protect worker health, specifically the health of working women. Public health messaging around the associated health risks of exposure to workplace harassment, particularly if it emphasizes potential costs to employers in the form of increased healthcare costs and possible loss of productivity due to employee absences, may help prompt employers to strengthen prevention efforts. Unfortunately, in the US, there is no federal law that prohibits generalized harassment or workplace bullying. Our results suggest that establishing and/or enforcing state or, preferably, federal anti-harassment policies or laws may help prevent unnecessary deaths associated with exposure to workplace harassment, particularly among women.

## Figures and Tables

**Table 1.** Sample characteristics (*n* = 1745 initial respondents; 249 deaths).

	Black Females (*n* = 338)	White Females (*n* = 632)	Black Males (*n* = 162)	White Males (*n* = 613)
Deaths n (%)	52 (15.4)	65 (10.3)	40 (24.7)	92 (15.0)
Age at Baseline mean (SD)	41.5 (9.6)	41.0 (11.5)	42.7 (11.3)	42.7 (13.0)
Occupational Group *n* (%)				
Clerical/Administrative	227 (67.2)	103 (16.3)	45 (27.8)	38 (6.2)
Faculty	20 (5.9)	288 (45.6)	9 (5.5)	305 (50.3)
Graduate Students	28 (8.3)	225 (35.6)	12 (7.4)	221 (36.0)
Service/Maintenance	63 (18.6)	16 (2.5)	96 (59.3)	46 (7.5)
Sexual Harassment (SH) at Baseline mean (SD)	21.2 (4.3) (*n* = 310)	21.3 (3.7) (*n* = 597)	22.5 (5.6) (*n* = 150)	20.8 (3.1) (*n* = 578)
Generalized Harassment (GH) at Baseline mean (SD)	40.6 (12.4) (*n* = 299)	37.3 (9.3) (*n* = 561)	41.3 (12.7) (*n* = 149)	36.9 (9.2) (*n* = 549)

Note: The *n* values for sexual harassment and generalized harassment differ from the total *n* for each group due to cases missing data on the harassment variables.

**Table 2.** Proportional hazards models for risk of death: conditional effects of time-dependent workplace harassment variables, sexual harassment (SH) and generalized harassment (GH), adjusted for occupational group and current age.

Hazard Ratio (*p*-Value) [95% Confidence Interval]	Black Females	White Females	Black Males	White Males
Effect of Current SH (*n* = 1635; 228 deaths)	1.048 (0.0889) [0.993, 1.107]	0.957 (0.4722) [0.847, 1.080]	0.927 (0.2487) [0.816, 1.054]	0.960 (0.4791) [0.858, 1.074]
Effect of Current GH (*n* = 1558; 211 deaths)	1.022 (0.1032) [0.996, 1.050]	1.033 (0.0027) [1.011, 1.055]	0.996 (0.8358) [0.963, 1.031]	1.001 (0.9400) [0.973, 1.030]

## Data Availability

The raw data supporting the conclusions of this article will be made available by the authors on request to the corresponding author.

## Appendix A

**Table A1.** Proportional hazards models for risk of death: conditional effects of time-dependent variables drinks per day (DRINKS), alcohol misuse (BMAST), and depressive symptoms (CESD), adjusted for occupational group and current age.

Hazard Ratio (*p*-Value) [95% Confidence Interval]	Black Females	White Females	Black Males	White Males
Effect of Current DRINKS (N = 1744; 247 deaths)	0.967 (0.8093) [0.739, 1.266]	0.569 (0.0008) [0.410, 0.792]	1.018 (0.8554) [0.837, 1.239]	1.000 (0.9998) [0.840, 1.191]
Effect of Current BMAST (N = 1790; 252 deaths)	0.880 (0.1219) [0.748, 1.035]	1.023 (0.7995) [0.861, 1.215]	1.033 (0.4137) [0.955, 1.117]	1.1410 (<0.0001) [1.068, 1.219]
Effect of Current CESD (N = 1715; 229 deaths)	1.066 (0.0473) [1.001, 1.136]	1.050 (0.0945) [0.992, 1.112]	1.082 (0.0256) [1.010, 1.160]	1.023 (0.4831) [0.961, 1.088]

Note: BMAST = Brief Michigan Alcoholism Screening Test; CESD = 7 items from the Center for Epidemiological Studies Depression scale.

**Table A2.** Proportional hazards models for risk of death: independent effects of GH and drinking (DRINKS), alcohol misuse (BMAST), or depressive symptoms (CESD), adjusted for occupational group and current age.

Hazard Ratio (*p*-Value) [95% Confidence Interval]	Black Females	White Females	Black Males	White Males
GH and DRINKS (N = 1509; 204 deaths)				
Effect of Current GH	1.020 (0.1605) [0.992, 1.049]	1.028 (0.0151) [1.005, 1.051]	0.995 (0.7747) [0.962, 1.029]	1.003 (0.8219) [0.976, 1.032]
Effect of Current DRINKS	0.978 (0.8779) [0.733, 1.303]	0.539 (0.0011) [0.372, 0.781]	1.127 (0.3759) [0.865, 1.467]	0.994 (0.9225) [0.825, 1.191]
GH and BMAST (N = 1551; 209 deaths)				
Effect of Current GH	1.021 (0.1197) [0.995, 1.049]	1.034 (0.0022) [1.012, 1.057]	0.997 (0.8429) [0.963, 1.032]	0.995 (0.7235) [0.995, 1.049]
Effect of Current BMAST	0.892 (0.2031) [0.747, 1.064]	1.026 (0.7849) [0.851, 1.237]	1.056 (0.1739) [0.975, 1.143]	1.161 (<0.001) [1.085, 1.243]
GH and CESD (N = 1490; 193 deaths)				
Effect of Current GH	1.021 (0.1656) [0.992, 1.051]	1.034 (0.0034) [1.011, 1.057]	0.984 (0.4044) [0.949, 1.021]	1.002 (0.8777) [0.974, 1.031]
Effect of Current CESD	1.067 (0.0700) [0.995, 1.145]	1.032 (0.3451) [0.967, 1.102]	1.087 (0.0321) [1.007, 1.173]	1.069 (0.0326) [1.005, 1.136]

Note: GH = generalized workplace harassment; BMAST = Brief Michigan Alcoholism Screening Test; CESD = 7 items from the Center for Epidemiological Studies Depression scale.

**Table A3.** Proportional hazards models for risk of death: independent effects of SH and drinking (DRINKS), alcohol misuse (BMAST), or depressive symptoms (CESD), adjusted for occupational group and current age.

Hazard Ratio (*p*-Value) [95% Confidence Interval]	Black Females	White Females	Black Males	White Males
SH and DRINKS (N = 1588; 221 deaths)				
Effect of Current SH	1.047 (0.1041) [0.991, 1.107]	0.936 (0.3915) [0.806, 1.088]	0.922 (0.2295) [0.809, 1.052]	0.965 (0.5272) [0.863, 1.078]
Effect of Current DRINKS	0.965 (0.8105) [0.721, 1.291]	0.561 (0.0011) [0.364, 0.794]	1.134 (0.2863) [0.900, 1.429]	1.043 (0.6288) [0.878, 1.240]
SH and BMAST (N = 1628; 226 deaths)				
Effect of Current SH	1.044 (0.1247) [0.988, 1.103]	0.952 (0.4384) [0.842, 1.077]	0.926 (0.2470) [0.812, 1.055]	0.950 (0.3923) [0.844, 1.069]
Effect of Current BMAST	0.913 (0.3515) [0.753, 1.106]	1.035 (0.7011) [0.867, 1.237]	1.035 (0.3942) [0.957, 1.119]	1.146 (<0.001) [1.071, 1.226]
SH and CESD (N = 1564; 205 deaths)				
Effect of Current SH	1.055 (0.0905) [0.992, 1.122]	0.927 (0.2783) [0.809, 1.063]	0.897 (0.1170) [0.783, 1.028]	0.962 (0.4973) [0.859, 1.077]
Effect of Current CESD	1.077 (0.0339) [1.006, 1.154]	1.060 (0.0632) [0.997, 1.126]	1.092 (0.0176) [1.015, 1.174]	1.041 (0.2195) [0.977, 1.109]

Note: SH = workplace sexual harassment; BMAST = Brief Michigan Alcoholism Screening Test; CESD = 7 items from the Center for Epidemiological Studies Depression scale.
